# FINDING THE ELDERS WHO STAYED- CONDUCTING OUTREACH IN THE AFTERMATH OF HURRICANE MICHAEL

**DOI:** 10.1093/geroni/igz038.3431

**Published:** 2019-11-08

**Authors:** Jessica L Tice, Megan Bond

**Affiliations:** Florida Department of Elder Affairs, Tallahassee, Florida, United States

## Abstract

The Florida Department of Elder Affairs (DOEA) provides programs and services for over 65,300 older people and adults with disabilities. These individuals are uniquely vulnerable and may be displaced, and/or disoriented during natural disasters. DOEA clients are dependent upon community-based services to provide supervision or assistance to perform basic self-care, which often makes sheltering in place alone a danger to their health and well-being. During Hurricane Michael (2018) many older adults who previously were independent sought help for many issues including property damage, utility interruption, food and medicine scarcity, and physical or mental health problems associated with the storm and its aftermath. In normal conditions, DOEA identifies older populations via Census tracts and then conducts outreach events to inform the public how to access social services. However, after the widespread displacement post-storm, traditional outreach approaches were insufficient. A method was needed to remove areas that were rendered uninhabitable and find who remained in place. DOEA identified viable neighborhoods by overlaying property damage locations on base layers of Census tracts with concentrations of older adults and polling places with high percentage of age 60+ voter participation in the subsequent November election. Then in partnership with Feeding Florida, we provided information and registration assistance via local food distribution sites in those areas. This methodology of overlaying Division of Emergency Management property damage records and voter participation records against publicly available Census tract files is a strategy that could be replicated by other disaster and flood-prone communities or organizations that have similar needs.

